# A Position-Specific, Time-Efficient Warm-Up Injury Prevention Program in Male Football Players: A Season-Long Intervention Study

**DOI:** 10.3390/sports14080340

**Published:** 2026-08-07

**Authors:** Dimitrios Vlachodimos, Athanasios A. Dalamitros, Vasiliki Manou, Thomas Metaxas, Nikolaos Stavropoulos, Ioannis Terzidis

**Affiliations:** Faculty of Physical Education and Sport Sciences, Aristotle University of Thessaloniki, Thermi, 57001 AUTH DPESS Thessaloniki, Greece; dvlachod@phed.auth.gr (D.V.); vmanou@phed.auth.gr (V.M.); tommet@phed.auth.gr (T.M.); nstavrop@phed.auth.gr (N.S.); terzidis@phed.auth.gr (I.T.)

**Keywords:** injury prevention, playing position, warm-up, football

## Abstract

This study assessed the effectiveness of a position-specific and time-efficient warm-up injury prevention program for male football players. This season-long quasi-experimental study included 129 male amateur football players. A structured injury prevention program was incorporated into the warm-up routine during the preseason and competitive season. Prospectively collected injury data from the intervention season were compared with retrospectively collected injury data from the previous season and included injury incidence, the number of injured players, injury severity, and injury burden. The program included general exercises and position-specific modifications for defenders, midfielders, and attackers. Implementation of the program was associated with a 60% reduction in total injuries (*p* < 0.05) and a 60.3% reduction in total injured players The injury burden was significantly reduced (*p* < 0.05), as indicated by a reduced mean day lost per injury from 33.24 to 19.56 days. Although injury reductions were observed across all playing positions, between-position differences were not statistically significant. The type of injuries and their mechanisms were not significantly affected. These preliminary, associative findings suggest that a time-efficient, position-specific warm-up program may contribute to reduced injury occurrence in amateur football; however, given the quasi-experimental design, confirmation through randomized controlled trials is required.

## 1. Introduction

Football is one of the most widely practiced sports worldwide, with millions of registered players participating at both amateur and professional levels [1]. Despite its global popularity, football is associated with a substantial risk of injury, which negatively affects player availability, team performance, and long-term athlete health [2,3]. Lower-limb injuries, particularly those affecting the hamstrings, knee (including anterior cruciate ligament [ACL]), groin, and ankle, are consistently reported as the most prevalent in male football players across competitive levels [4]. These injuries often result in significant time loss and contribute disproportionately to overall injury burden [5].

A large body of evidence indicates that many football-related injuries are at least partially preventable [6]. In this direction, exercise-based injury prevention programs incorporating strength, neuromuscular control, balance, and plyometric components have been shown to reduce injury occurrence, particularly when compliance is high [7]. For instance, eccentric hamstring strengthening has been associated with substantial reductions in hamstring injuries [8], while neuromuscular and proprioceptive training programs have demonstrated effectiveness in reducing knee and ankle injuries [9]. Comprehensive warm-up programs, such as the FIFA 11+, have further shown that structured, multi-component interventions can reduce overall injury risk in football populations [10].

Despite this evidence, the widespread implementation of injury prevention programs in real-world settings remains limited. One of the most frequently reported barriers is time constraint, as coaches are often reluctant to allocate more than 10 to 15 min of training time to preventive exercises [11]. Additional barriers include lack of individualization and supervision, perceived complexity of programs, small sample sizes, low compliance with the program, its limited integration into regular training routines, and a failure to account for pre-existing injuries and implement systematic progression [12]. Hence, there is a need for injury prevention strategies that are evidence-based and time-efficient, while in parallel, practical, and easily integrated into standard warm-up routines. Moreover, recent studies further support the value of soccer-specific training interventions that are both practical and engaging. Game-representative training methods, including small-sided games and reactive agility exercises, have been shown to improve physical performance while enhancing player enjoyment and adherence, highlighting the importance of designing injury-prevention programs that are not only effective but also feasible and motivating within regular football training environments [13,14].

Another important yet underexplored consideration is the role of playing position in injury risk and prevention. In football, different playing positions are exposed to distinct physical and tactical demands, including variations in sprinting, acceleration, deceleration, changes in direction, and contact situations [15,16]. These differences are associated with position-specific injury patterns, with evidence suggesting that midfielders may be more prone to hamstring injuries [17], defenders to contact-related injuries [18], and attackers to high-speed and explosive actions [19]. While these positional differences are well documented, most existing injury prevention programs tend to adopt a “one-size-fits-all” approach, without tailoring exercises to the specific demands of each playing role. Moreover, as physical and tactical demands differ substantially among defenders, midfielders, and attackers, injury risk profiles may also differ, potentially warranting tailored preventive strategies.

To date, limited research has examined whether incorporating position-specific adaptations into a structured, time-efficient warm-up program can enhance injury prevention outcomes [9]. Furthermore, few studies have evaluated such interventions over an entire competitive season in amateur football players, where implementation challenges are often greatest but potential benefits are substantial [20]. To our knowledge, no previous study has evaluated a time-efficient, position-specific injury prevention program over a full competitive season in amateur male football players. Therefore, the present study aimed to evaluate the effectiveness of a position-specific, time-efficient (<15 min) warm-up injury prevention program, implemented twice weekly over a full competitive season, in reducing injury occurrence and injury burden in male amateur football players. We hypothesized that the program would significantly reduce injury occurrence, the number of injured players, and injury burden compared with the previous season.

## 2. Materials and Methods

### 2.1. Study Design

This study employed a quasi-experimental prospective before–after design comparing injury outcomes during a retrospective pre-intervention season and a prospective intervention season. In May 2023, coaches and team representatives from nine amateur football clubs in Greece were contacted and informed about the study procedures, as injury prevention programs recommended to commence at least six weeks prior to the start of the season followed by a maintenance program and be part of the warm-up routine [21]. Six teams agreed to participate, while three teams declined. In total, 129 male amateur football players participated in the study from August 2023 to May 2024. Inclusion criteria required players to (a) be registered members of their team during both the preseason and competitive season, (b) participate in team training at least three times per week, and (c) be available for official matches. Players who had not participated in the previous competitive season, goalkeepers, and players younger than 16 years of age were excluded.

**Figure 1:**
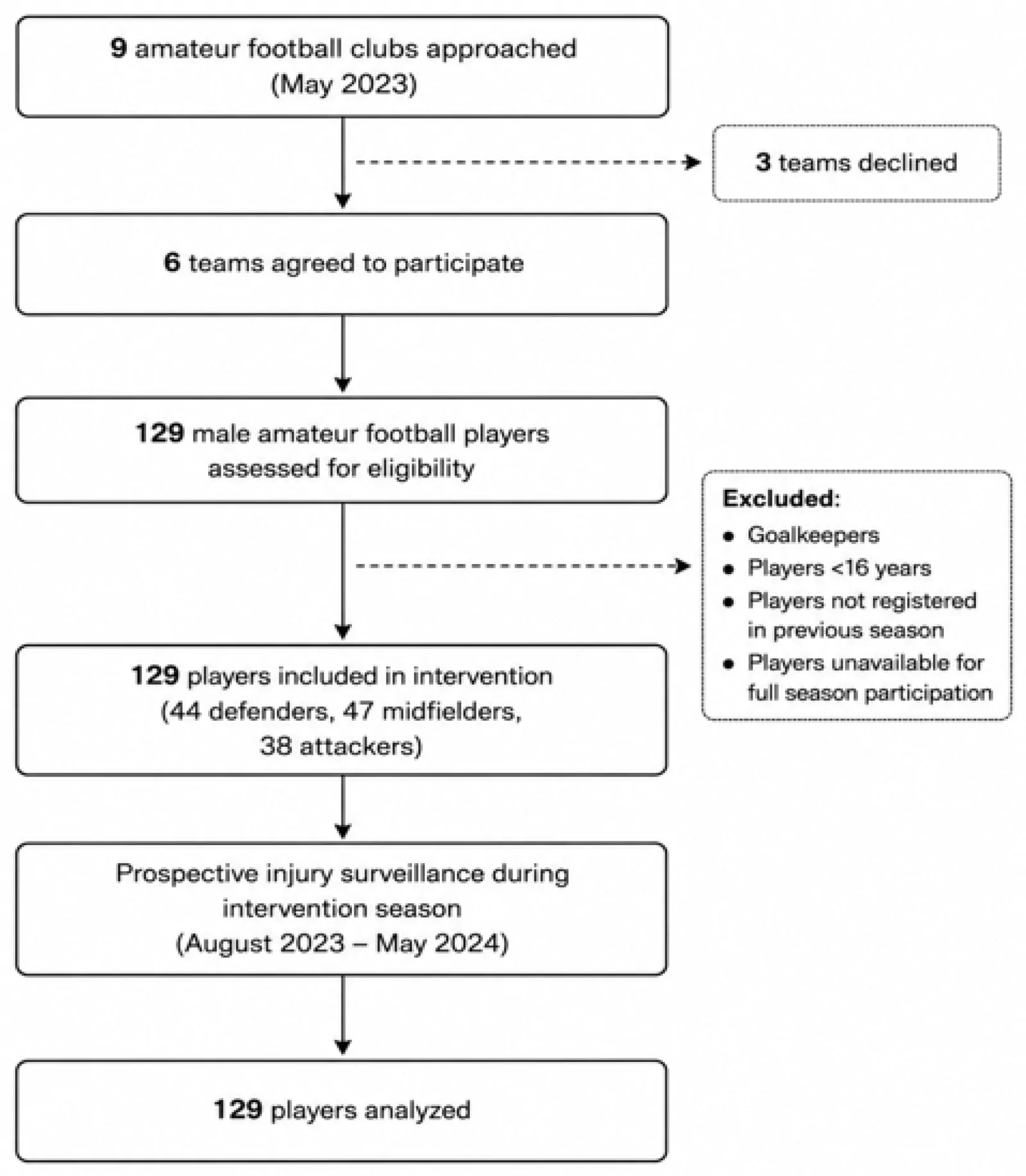
Flow diagram of participant recruitment, eligibility assessment, inclusion, and analysis.

The study sample consisted of 129 male football players (44 defenders, 47 midfielders, and 38 attackers), aged 16–32 years (mean 19.47 ± 3.49), with a height of 163–197 cm (mean 179.43 ± 6.94), body mass of 49.5–95 kg (mean 72.46 ± 8.87), training age of 5–28 years (mean = 12.9 ± 3.81), and a training frequency of 2 to 6 sessions per week (mean 4.61 ± 0.70). Participant recruitment and study inclusion are summarized in Figure 1, while the baseline characteristics of the study participants are presented in Table 1.

During the first week of the preseason, each team received an on-field presentation of the injury prevention program. Written and visual instructional materials were provided to coaches, strength and conditioning staff, and players. Anthropometric data, playing experience, playing position, and injury history from the previous season were recorded at baseline. To ensure consistent implementation and high compliance, a research associate attended at least one training session per team each week. During each visit, the research associate monitored correct exercise execution, adherence to the prescribed program structure, and provided corrective feedback to players and coaching staff where necessary. Any deviations from the prescribed exercises, progressions, or session structure were noted and addressed at the subsequent visit. Injury data were systematically collected throughout the study period using a standardized surveillance protocol [22]. Previous research has shown that greater compliance with injury prevention programs is associated with larger reductions in injury risk [23]. The study design was approved by the Research Ethics Committee of the School of Physical Education and Sport Science at Thessaloniki (Approval number 151/2023). All participants were informed about the procedures approved by the ethics committee and in compliance with the Declaration of Helsinki and provided individual written informed consent; for participants under the age of 18, consent was obtained from their parents or legal guardians. Reporting of this study was informed by the TREND (Transparent Reporting of Evaluations with Nonrandomized Designs) statement for non-randomized intervention studies.

### 2.2. Data Collection and Injury Classification

Injury data from the season preceding the intervention were collected retrospectively, on a weekly basis. At baseline, the research associate conducted structured interviews with players and, where available, corroborated by coaches and team staff (strength and conditioning staff, and medical personnel, when available) prior to training sessions. To improve consistency of reporting, the same time-loss injury definition and injury classification criteria used during prospective surveillance were applied retrospectively. This approach minimized recall delay and improved reporting accuracy. Data collection focused on injury occurrence, anatomical location, injury mechanism, and time-loss duration. Injuries were defined as time-loss injuries, resulting in absence from training or match participation. Injury severity was classified as minimal (1–3 days), mild (4–7 days), moderate (8–21 days), or severe (>21 days), in accordance with established consensus guidelines [22]. Injuries resulting in no time loss were excluded due to inconsistent reporting reliability. No “career-ending” injuries were recorded during the study period. Injury data from the previous season to the intervention period was collected retrospectively at baseline via structured interviews. While this method has been shown to provide acceptable recall for injury occurrence and location, diagnostic accuracy may be reduced compared to prospective surveillance.

### 2.3. Intervention Program

The injury prevention program was designed to be time-efficient (≤15 min), equipment-free, and fully integrated into the regular team warm-up routine. It was performed twice per week throughout both the preseason and the competitive season. Throughout the intervention period, the program also involved systematic progression of the selected exercises at six-week intervals, along with position-specific modifications based on the participants’ playing roles. Progression involved increasing eccentric volume, shifting from bilateral to unilateral stability patterns, and accelerating the velocity of plyometric tasks. Finally, after the 12 weeks, the final level of the injury prevention program was established and then maintained without any modifications until the end of the study.

The injury prevention program was developed based on current scientific literature addressing the general and football-specific mechanisms of injury [24,25], relevant risk factors [26,27] and evidence-based prevention strategies [28,29] for hamstring, knee/ACL, adductor, and ankle sprain injuries. In addition, expert knowledge derived from highly experienced practitioners working at the highest level of football conditioning, as well as practical input from players, coaches, strength and conditioning coaches, and physiotherapists from professional football clubs [8], was incorporated into the program design. The primary outcome variables were total injuries, number of injured players, and injury burden. Secondary outcomes included injury severity, injury type, and injury mechanism. Positional analyses were conducted for defenders, midfielders, and attackers. The intervention consisted of a position-specific, progressive warm-up program performed twice weekly for the duration of the season. The structure of the program consisted of three distinct parts: Part A: General activation, strength, and neuromuscular control exercises performed by all players; Part B: Position-specific exercises targeting predominant injury risk profiles associated with defenders, midfielders, and attackers; Part C: An integrated football-specific drill including agility, change in direction, and high-speed running performed by all players. Detailed exercise descriptions and progressions are provided in Table 2.

### 2.4. Statistical Analysis

Statistical analyses were performed using IBM SPSS Statistics (Version 29.0). Categorical variables, including injury occurrence, number of injured players, injury severity, injury type, and injury mechanism, were analysed using chi-square tests. Mean differences in days lost due to injury were assessed using independent sample *t*-tests. To complement null-hypothesis significance testing, effect sizes were calculated to quantify the practical magnitude of observed differences. Risk ratios (RR) were used to assess relative changes in injury occurrence between seasons. Although exposure-adjusted incidence rate ratios are preferable when player-hours are available, RR was selected because exposure data were not recorded. Cohen’s d was applied to evaluate standardized differences in mean days lost due to injury, while Cramer’s V was used to estimate effect size for categorical comparisons involving injury severity, injury type, and injury mechanism.

This combined approach was adopted to enhance interpretability and to account for the limited statistical power inherent in subgroup analyses. Effect sizes were interpreted according to established conventions [30,31]. Cohen’s d values below 0.20 were considered trivial, and Cohen’s d values of 0.20, 0.50, and 0.80 were considered small, moderate, and large, respectively, while Cramer’s V values of 0.10, 0.30, and 0.50 indicated small, moderate, and large effects. For RR, interpretation was based on the magnitude of relative risk reduction, in line with applied epidemiological practice and recommendations emphasizing practical significance beyond *p*-values [32]. Statistical significance was set at *p* < 0.05.

## 3. Results

### 3.1. Number of Injuries

Following the implementation of the position-specific warm-up injury prevention program, a substantial reduction in overall injuries was observed. The total number of recorded injuries decreased from 80 in the season prior to the intervention to 32 during the intervention season, corresponding to a 60% reduction (Table 2). This decrease was statistically significant (χ^2^ = 4.949, df = 1, *p* = 0.026) and was associated with a large practical effect RR = 0.40; relative risk reduction = 60%). When examined by playing position, injury reductions were observed across all positional groups. Defenders sustained 13 injuries during the intervention season compared to 20 in the previous season (35% reduction), midfielders sustained 12 injuries compared to 30 (60% reduction), and attackers sustained 7 injuries compared to 30 (76.7% reduction) (Table 3). Although these reductions did not reach statistical significance at the positional level, the magnitude of change was descriptively largest for attackers; this should be interpreted cautiously given the lack of statistical significance and the limited subgroup sample sizes.

### 3.2. Number of Injured Players

The number of injured players was also markedly reduced following the intervention. A total of 68 players sustained at least one injury in the season prior to the intervention, compared to 27 players during the intervention season, representing a 60.3% reduction. This corresponds to a large effect size (RR = 0.40). At the positional level, the number of injured defenders decreased from 18 to 11 (38.9% reduction), midfielders from 24 to 10 (58.3% reduction), and attackers from 26 to 6 (76.9% reduction) (Table 4). While these changes were not statistically significant, the direction and magnitude of the effects were consistent across positions. Table 4 presents the total number of injured athletes and the number of injured athletes by playing position.

### 3.3. Injuries by Playing Position

Across all positional subgroups, the implementation of the prevention program was accompanied by a clear downward trend in both total injury incidence and the absolute number of injured players.

#### 3.3.1. Defenders

The number of injured defenders decreased from 18 to 11 following the intervention (38.9% reduction) (Table 4), although this change was not statistically significant (*p* = 0.723). However, injury severity among defenders was significantly reduced (*p* = 0.006) (Table 5b).

#### 3.3.2. Midfielders

Among midfielders, the number of injured players decreased from 24 to 10 (58.3% reduction) (Table 4), though this reduction did not reach statistical significance (*p* = 0.430). No significant change in injury severity was observed (*p* = 0.621) (Table 5b).

#### 3.3.3. Attackers

Attackers demonstrated the largest relative reduction in injuries, with injured players decreasing from 26 to 6 (76.9% reduction) (Table 4). This change was not statistically significant (*p* = 0.920), and no significant difference in injury severity was observed (*p* = 0.780) (Table 5b).

### 3.4. Injury Burden and Severity

Injury burden, assessed through injury severity categories and time-loss days, was significantly reduced following the implementation of the injury prevention program (Table 5). The distribution of injury severity differed significantly between seasons (χ^2^ = 8.113, df = 3, *p* = 0.044), with a moderate effect size (Cramer’s V = 0.27). Severe injuries (>21 days absence) showed the largest relative reduction, decreasing from 27 cases before the intervention to 4 cases during the intervention season, representing an 85.2% reduction (Table 5a). This change reflects a large practical effect, reflecting a substantial reduction in high-burden injuries. Mean days lost per injury also decreased from 33.24 ± 50.18 days before the intervention to 19.56 ± 32.56 days after the intervention (mean difference = 13.68 days; 95% CI: −5.35 to 32.70) (Table 6). Although this reduction did not reach statistical significance (t = 1.424, df = 110, *p* = 0.157), the effect estimate indicated a small-to-moderate positive effect (Cohen’s d = 0.34; 95% CI: −0.07 to 0.75) [30,31]; however, the confidence interval crossed zero, indicating uncertainty regarding the precision and direction of the true effect. As no clinical threshold for meaningful change was predefined in this study, this finding should be interpreted descriptively rather than as evidence of a clinically relevant trend.

### 3.5. Injury Type and Mechanism

The distribution of injury types did not differ significantly between seasons (*χ*^2^ = 2.175, *df* = 4, *p* = 0.704), with a small effect size (Cramer’s V = 0.12). Hamstring strains were the most common specific injury in both the pre-intervention and intervention seasons, accounting for 18.8% of all injuries in each period (15 vs. 6 injuries). Ankle sprains decreased from 15 to 4 cases, while adductor strains decreased from 6 to 5 cases. Knee/ACL injuries decreased from 4 to 2 cases. Injuries classified as other injuries (e.g., contusions, muscle cramps, lacerations) declined from 40 to 15 cases. Despite these descriptive reductions, the overall distribution of injury types did not differ significantly between seasons (χ^2^ = 2.175, *p* = 0.704).

Finally, injury mechanisms demonstrated similar distributions before and after the intervention. Injuries occurring during changes in direction decreased from 15 to 4 cases, while injuries associated with kicking or passing decreased from 14 to 4 cases. Tackling- or stretching-related injuries were reduced from 10 to 1 case. Running-related injuries showed minimal change (9 vs. 8 cases), whereas overload or unknown mechanisms decreased from 12 to 8 cases. Although several mechanisms showed descriptive reductions, the overall distribution of injury mechanisms was not significantly different between seasons (χ^2^ = 7.032, *p* = 0.318).

## 4. Discussion

To our knowledge, this is among the first studies evaluating a position-specific, time-efficient (<15 min) football warm-up injury prevention intervention in male amateur football players. The main findings support the primary hypothesis, demonstrating a substantial reduction in overall injury occurrence, number of injured players, and injury burden following the implementation of the intervention, suggesting that a structured warm-up program lasting less than 15 min and incorporating position-specific adaptations may be associated with meaningful reductions in real-world football settings.

A key result of the present study was the approximately 60% reduction in total injuries and injured players during the intervention season. While this magnitude of change is statistically significant and practically substantial and consistent with previous literature on exercise-based injury prevention programs in football [33], it is important to emphasize that the quasi-experimental before–after design of the present study does not allow for causal attribution of this reduction to the intervention [34]. Direct comparisons with earlier studies, such as those evaluating general warm-up interventions [35], should also be made with caution due to differences in study design, populations, and outcome measures. The present findings are therefore best interpreted as preliminary associative evidence suggesting that a time-efficient, position-specific warm-up program may be associated with lower injury occurrence in amateur football, an observation that warrants confirmation through adequately powered randomized controlled trials.

In addition to reducing injury occurrence, the intervention was associated with a significant reduction in injury burden, as reflected by both severity distribution and mean days lost per injury. Notably, severe injuries (>21 days absence) were reduced, reflecting a shift toward less severe injury outcomes. From a performance and health perspective, this finding is particularly relevant, as injury burden is more strongly associated with player availability and team performance than injury occurrence alone [2]. One possible, though untested, explanation is that the intervention enhanced neuromuscular control, movement efficiency, or load tolerance [22]; since none of these variables were directly assessed, this remains entirely speculative.

The secondary hypothesis regarding positional differences was partially supported. Reductions in injury occurrence were observed across all positional groups, although the magnitude of change varied. This finding is consistent with the multifactorial nature of football injuries, which are influenced by a complex interaction of intrinsic and extrinsic factors [34]. Rather than targeting a single injury mechanism, the program may have contributed to improvements in general physical preparedness and resilience, thereby reducing susceptibility to a range of injury types. More specifically defenders demonstrated the smallest relative reduction (38.9%), as this playing position is related to more unpredictable contact-driven, collision-based injuries (tackling, aerial duels) [36]. Interestingly, ankle injuries were less frequent among defenders at baseline than would be expected based on previous reports in amateur football populations, which have generally identified defenders as being at elevated risk for ankle injuries [18,37]. Midfielders demonstrated a 58.3% reduction in injured players and a 33.3% reduction in hamstring injuries, consistent with evidence suggesting that midfielders may be particularly vulnerable to hamstring-related injuries due to their high running demands [14,17]. Attackers demonstrated the largest relative reduction (76.9%), accompanied by reductions in adductor strains, hamstring injuries, and ankle sprains, as well as no recorded ACL injuries during the intervention season. Attackers routinely execute unexpected high-speed cutting maneuvers and decelerations [19]. These descriptive trends should be regarded as preliminary and exploratory only. Because between-position differences were not statistically significant and subgroup sample sizes were limited, they should not be interpreted as evidence of differential program effectiveness across playing positions. Whether the position-specific exercise adaptations were differentially aligned with the movement demands [38] and injury profiles of each playing role [39] in a way that produced meaningful benefit cannot be determined from the present data and requires investigation in adequately powered future studies.

The position-specific structure of the program was designed to reflect differences in movement demands and injury profiles. The descriptive trends observed in the data are consistent with this rationale; however, given the absence of statistical significance in between-position comparisons and the small subgroup sample sizes, these trends should not be taken as confirmation of position-specific program effects.

This study has several important strengths. First, it demonstrates high ecological validity, as the intervention was fully integrated into the regular warm-up routine and implemented under real-world conditions in amateur football teams. Second, the program was time-efficient (<15 min), equipment-free, and easily scalable, directly addressing key barriers to implementation and enhancing its practical applicability. Third, the study introduced a novel position-specific approach, reflecting the differing physical demands of playing roles, and evaluated its effectiveness over an entire competitive season. In addition, the prospective injury surveillance during the intervention period, use of standardized injury definitions, and inclusion of effect size measures alongside statistical testing strengthen the interpretability and applied relevance of the findings.

### 4.1. Practical Implications

From a practical standpoint, the findings suggest that injury prevention strategies do not necessarily require extensive training time or specialized equipment to be effective. Position-specific adaptations incorporated into short warm-up routines may represent a feasible approach for amateur football environments, where limited resources and time constraints often reduce implementation of established prevention programs. Nevertheless, these findings should be considered preliminary until confirmed by adequately powered randomized controlled trials.

### 4.2. Limitations

This study has some limitations that should be acknowledged. First, this study employed a quasi-experimental before–after design without a concurrent control group, which limits causal inference and increases susceptibility to confounding factors such as seasonal variation, changes in training practices, or regression to the mean. Second, injury data from the pre-intervention season were collected retrospectively and therefore may be affected by recall bias and differences in reporting accuracy relative to the prospective surveillance applied during the intervention season. Although structured interviews and standardized injury definitions were used, retrospective self-reporting may underestimate minor injuries and reduce diagnostic precision. Consequently, comparisons between seasons should be interpreted cautiously and viewed as associative rather than causal. Third, exposure data (e.g., training and match hours) were not recorded, preventing the calculation of exposure-adjusted injury incidence (injuries per 1000 player-h). Thus, the observed reductions should be translated as changes in injury occurrence within this cohort rather than formal incidence-rate reductions. Fourth, although the presence of a research associate at weekly training sessions facilitated implementation monitoring and ensured a minimum level of program fidelity, compliance was not quantitatively assessed using validated measurement tools (e.g., session logs, attendance records, or exercise completion checklists). Consequently, the precise degree to which individual players and teams adhered to the prescribed program throughout the intervention season remains unknown. Finally, subgroup analyses by playing position were based on relatively small sample sizes, reducing statistical power to detect significant differences. Future randomized controlled trials should compare position-specific and non-position-specific prevention programs while accounting for exposure, compliance, training load, and previous injury history.

## 5. Conclusions

In conclusion, implementation of a position-specific, time-efficient warm-up program was associated with reduced injury occurrence and lower injury burden among male amateur football players over a competitive season. However, given the quasi-experimental before–after design without a concurrent control group, these findings are associative in nature and should not be interpreted as evidence of a causal program effect. Alternative explanations, including seasonal variation, regression to the mean, and uncontrolled confounders, cannot be excluded. Positional subgroup findings were exploratory and not statistically significant between groups. These observations suggest that short, easily implemented prevention strategies may be a feasible option in amateur football settings and provide a foundation for future randomized controlled trials to determine the independent contribution of position-specific programming to injury reduction.

## Figures and Tables

**Table 1:** Baseline characteristics of the study participants.

Variable	Total (n = 129)	Defenders (n = 44)	Midfielders (n = 47)	Attackers (n = 38)	*p* Value
Age (years)	19.47 ± 3.49	19.61 ± 3.27	19.49 ± 3.94	19.29 ± 3.16	0.916
Height (cm)	179.43 ± 6.94	179.58 ± 6.98	178.32 ± 6.48	180.63 ± 5.05	0.240
Body mass (kg)	72.46 ± 8.87	72.27 ± 8.49	71.12 ± 9.29	74.34 ± 8.66	0.247
Training age (years)	12.90 ± 3.81	12.64 ± 3.91	12.94 ± 4.02	13.16 ± 3.49	0.826
Weekly training frequency	4.61 ± 0.70	4.68 ± 0.74	4.62 ± 0.68	4.53 ± 0.69	0.607

**Table 2:** Detailed description of exercises, progressions, and position-specific modifications for the three parts of intervention injury prevention program.

**Part A: General Activation, Core, and Neuromuscular Control Components (All Players)**			
**Level 1**		**Level 2**	**Level 3**
2 min run		2 min run	2 min run
HW (4 × 40 cm)		HW (4 × 40 cm) increase tempo	HW (4 × 40 cm) increase tempo
SLDL (8 reps per leg)		SLDL (8 reps per leg)	SLDL with dynamic toe-touch (8 reps per leg)
4 SLFB + LJ per leg + 1 long jump on the opposite leg		4 SLFB + LJ + 1 long jump landing on the opposite leg	4 SLFB + LJ + 1 long jump landing on the opposite leg
SP (2 reps of 15 s)		SP inner leg kicks front and back with upper hand support (2 sets of 8 reps per leg)	SP inner leg kicks front and back without upper hand support (2 sets of 8 reps per leg)
BL with diagonal movements (8 reps per leg)		BL with diagonal and side movements (8 reps per leg)	BL with diagonal, side and front movements (8 reps per leg)
**Part B: Individualized, Position-Specific Neuromuscular Training**			
	**Level 1**	**Level 2**	**Level 3**
Defenders	SLDL (4 reps per leg)	SLDL (4 reps per leg)	SLDL with dynamic toe-touch (4 reps per leg)
	BL with diagonal movements (4 reps per leg)	BL with diagonal and side movements (4 reps per leg)	BL with diagonal, side and front movements (4 reps per leg)
Midfielders	SLDL (4 reps per leg)	SLDL (4 reps per leg)	SLDL with dynamic toe-touch (4 reps per leg)
	1 SLFB + LJ per leg + 1 long jump on the opposite leg	1 SLFB + LJ + 1 long jump landing on the opposite leg	1 SLFB + LJ + 1 long jump landing on the opposite leg
Attackers	2 SLFB + LJ per leg + 1 long jump on the opposite leg	2 SLFB + LJ + 1 long jump landing on the opposite leg	2 SLFB + LJ + 1 long jump landing on the opposite leg
**Part C: Integrated Football-Specific Drill (All Players)**			
**Level 1**		**Level 2**	**Level 3**
3 SR of 6 m, 90° directional turn (left or right), 6 m run, 90° turn in the opposite direction and 25 m high-speed sprint		3 SR of 6 m, 90° directional turn (left or right), 6 m run, 90° turn in the opposite direction and 25 m high speed sprint	3 SR of 6 m, 90° directional turn (left or right), 6 m run, 90° turn in the opposite direction and 25 m high-speed sprint

Note: HW = Hurdle walks; SLDL = Single-Leg Romanian Deadlift; SLFB = single-leg front-back; SP = Side plan; BL = Balance drill; LJ = Lateral Jumps; SR = shuttle runs.

**Table 3:** Number of recorded injuries before and after the intervention.

Playing Position	Pre-Intervention Injuries (n)	Post-Intervention Injuries (n)	Change (%)	Risk Ratio (95% CI)	*p* Value	Interpretation
Total	80	32	60.0	0.40 (0.27–0.59)	0.026	Large
Defenders	20	13	35.0	0.65 (0.34–1.12)	0.351	Uncertain
Midfielders	30	12	60.0	0.40 (0.22–0.71)	0.095	Large
Attackers	30	7	76.7	0.23 (0.11–0.47)	0.427	Large

*Note*: for the Attackers row, one cell had an expected count below 5 (minimum expected count = 2.12); Fisher’s exact test gave a consistent result (*p* = 0.655).

**Table 4:** Number of recorded injured players before and after the intervention.

Playing Position	Pre-Intervention Injured Players (n)	Post-Intervention Injured Players (n)	Change (%)	Risk Ratio (95% CI)	*p* Value	Interpretation
Total	68	27	60.3	0.40 (0.26–0.60)	0.333	Large
Defenders	18	11	38.9	0.61 (0.32–1.16)	0.723	Uncertain
Midfielders	24	10	58.3	0.42 (0.22–0.79)	0.430	Large
Attackers	26	6	76.9	0.23 (0.10–0.52)	0.920	Large

**Table 5:** (**a**) Injury severity before and after the intervention. (**b**) Injury severity by playing position, before and after the intervention.

**(a)**							
**Severity Category**		**Pre-Intervention Injuries (n)**		**Post-Intervention Injuries (n)**		**Change (%)**	
Minimal (1–3 days)		16		4		75	
Mild (4–7 days)		15		11		26.7	
Moderate (8–21 days)		22		13		40.9	
Severe (>21 days)		27		4		85.2	
(**b**)							
**Position**	**Minimal (1–3 d) Pre/Post**	**Mild (4–7 d) Pre/Post**	**Moderate (8–28 d) Pre/Post**	**Severe (28+ d) Pre/Post**	**χ^2^**	**df**	***p*** **Value**
Defenders	7/1	2/4	1/5	8/1	12.305	3	0.006
Midfielders	2/2	6/1	10/5	6/2	1.774	3	0.621
Attackers	4/1	6/2	6/2	10/1	1.089	3	0.780

Overall comparison: χ^2^ = 8.113, df = 3, *p* = 0.044, Cramer’s V = 0.27. *Note*: because of the small subgroup sample sizes, most cells have an expected count below 5 for these position-specific severity comparisons (Defenders: 7/8 cells; Midfielders: 13/14 cells; Attackers: 12/14 cells); these results should be interpreted with caution.

**Table 6:** Injury burden before and after the intervention.

	Pre-Intervention	Post-Intervention	Mean Difference (95% CI)	*p* Value	Cohen’s d (95% CI)
Days lost per injury	33.24 ± 50.18	19.56 ± 32.56	13.68 (−5.35 to 32.70)	0.157	0.34 (−0.07 to 0.75)

## Data Availability

Research data are available from the corresponding author upon reasonable request.
